# Surgical hip dislocation for the management of acetabular osteoid osteoma in an adolescent: a case report

**DOI:** 10.1093/jscr/rjag572

**Published:** 2026-07-10

**Authors:** Stefan Tserovski, Nia Gecheva, Vensan Velchov, Georgi Lukanov, Atanas Panev, Alexander Gerchev

**Affiliations:** University Hospital of Orthopedics “Prof. B. Boichev”, Blvd. Nikola Petkova 56, Sofia, 1614, Bulgaria; Medical University Sofia, 15 Acad. Ivan Geshov Blvd., Sofia, 1431, Bulgaria; University Hospital of Orthopedics “Prof. B. Boichev”, Blvd. Nikola Petkova 56, Sofia, 1614, Bulgaria; Medical University Sofia, 15 Acad. Ivan Geshov Blvd., Sofia, 1431, Bulgaria; University Hospital of Orthopedics “Prof. B. Boichev”, Blvd. Nikola Petkova 56, Sofia, 1614, Bulgaria; Medical University Sofia, 15 Acad. Ivan Geshov Blvd., Sofia, 1431, Bulgaria; University Hospital of Orthopedics “Prof. B. Boichev”, Blvd. Nikola Petkova 56, Sofia, 1614, Bulgaria; Medical University Sofia, 15 Acad. Ivan Geshov Blvd., Sofia, 1431, Bulgaria; University Hospital of Orthopedics “Prof. B. Boichev”, Blvd. Nikola Petkova 56, Sofia, 1614, Bulgaria; Medical University Sofia, 15 Acad. Ivan Geshov Blvd., Sofia, 1431, Bulgaria; University Hospital of Orthopedics “Prof. B. Boichev”, Blvd. Nikola Petkova 56, Sofia, 1614, Bulgaria; Medical University Sofia, 15 Acad. Ivan Geshov Blvd., Sofia, 1431, Bulgaria

**Keywords:** osteoid osteoma, hip pathology, surgical hip dislocation

## Abstract

Osteoid osteoma of the acetabulum is a rare and diagnostically challenging condition that may mimic intra-articular hip pathology. We present the case of a 15-year-old boy with persistent nocturnal hip pain initially misdiagnosed as femoroacetabular impingement. Imaging with computed tomography and magnetic resonance imaging revealed a 1.4 cm nidus located in the central acetabular plate. The patient underwent surgical hip dislocation with complete excision of the lesion. Postoperatively, the patient became pain-free, and at 6-month follow-up showed full functional recovery. This case highlights the importance of considering osteoid osteoma in adolescents with atypical hip pain and the role of surgical hip dislocation for lesions in difficult anatomical locations.

## Introduction

Osteoid osteoma is a benign osteoblastic tumor that typically affects adolescents and young adults. It is characterized by nocturnal pain and a good response to nonsteroidal anti-Inflammatory drugs (NSAIDs). While most commonly located in the diaphysis of long bones, intra-articular and acetabular locations are rare and often lead to delayed or incorrect diagnosis due to atypical clinical and radiological presentation. Acetabular osteoid osteoma may mimic conditions such as femoroacetabular impingement or synovitis, making diagnosis challenging. Computed tomography (CT) remains the gold standard for identifying the nidus, while magnetic resonance imaging (MRI) findings may be misleading due to extensive bone marrow edema.

## Case presentation

A 15-year-old boy presented with persistent right hip pain, predominantly nocturnal, partially relieved by NSAIDs. He had previously undergone hip arthroscopy for presumed cam-type femoroacetabular impingement without symptomatic improvement.

On clinical examination, the patient demonstrated limping and pain exacerbated by physical activity.

**Figure 1:**
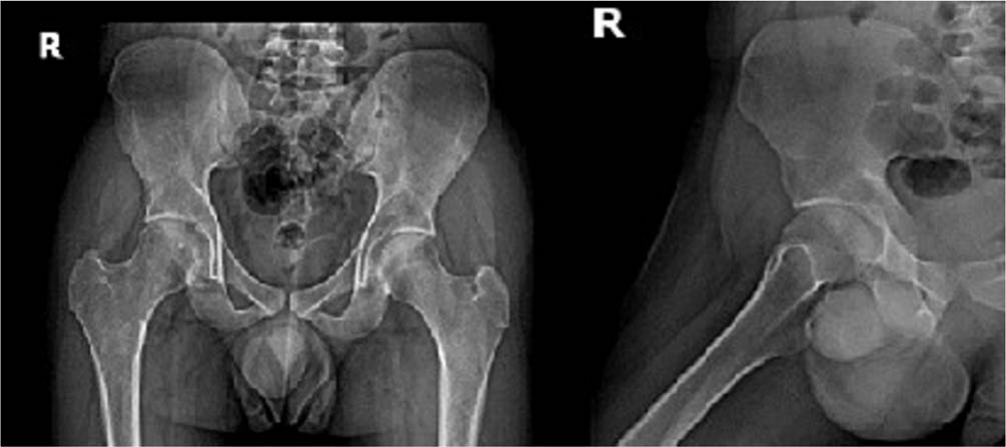
Imaging findings: Radiographs AP and lateral: Inconclusive findings of hip pathology.

**Figure 2:**
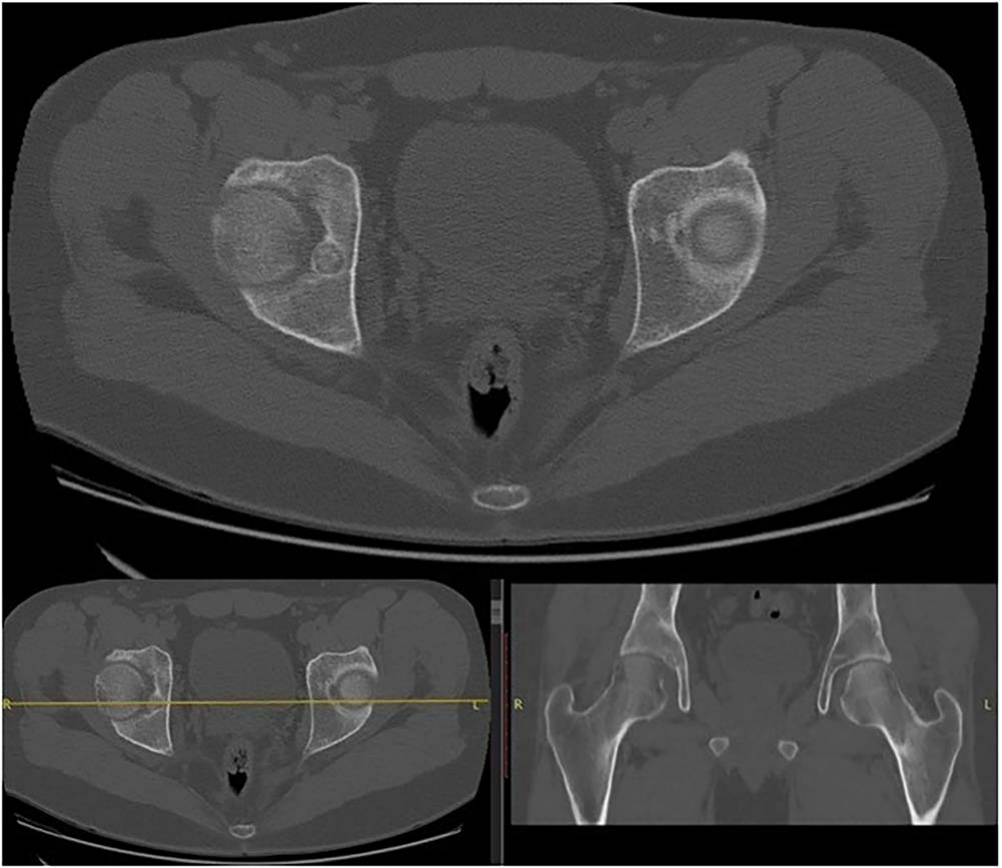
A well-defined 1.4 cm nidus with a surrounding sclerotic rim located in the central portion of the acetabular cancellous bone.

**Figure 3:**
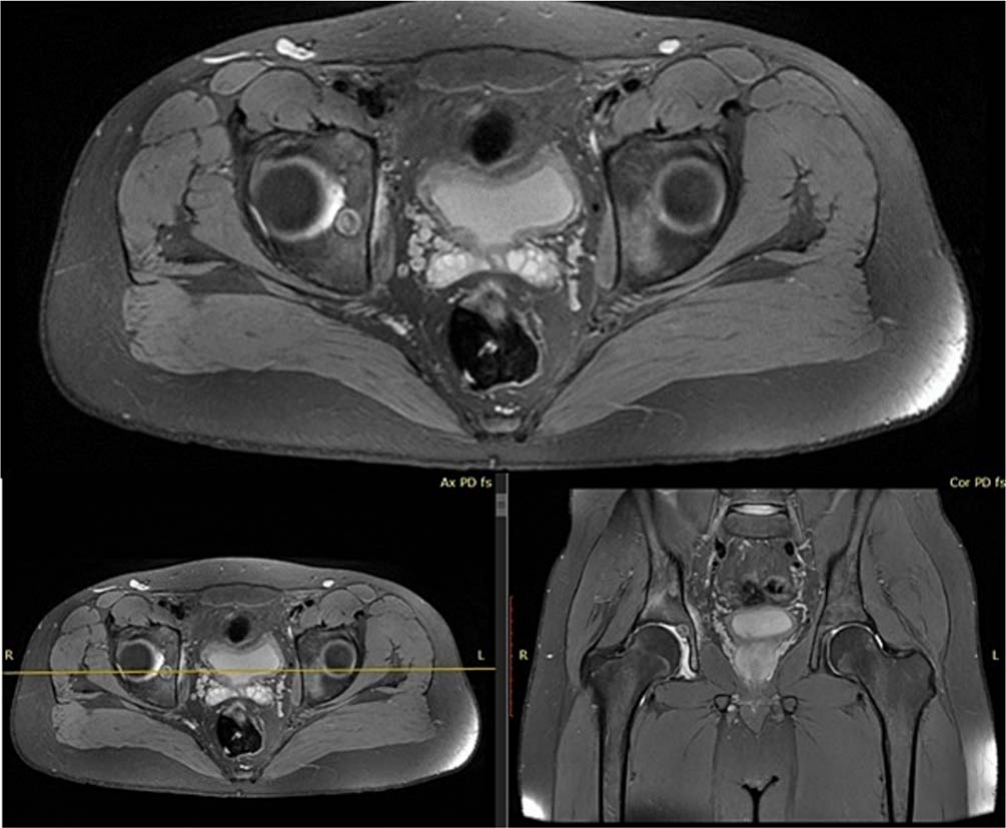
MRI findings supporting initial CT diagnosis.

Plain radiographs were inconclusive (Fig. 1). CT imaging demonstrated a well-defined 1.4 cm nidus with a surrounding sclerotic rim located in the central portion of the acetabular cancellous bone (Figs 2 and 3).

Due to the deep intra-articular location of the lesion, surgical hip dislocation was performed. The position of the nidus was determined preoperatively using CT measurements and confirmed intraoperatively with fluoroscopic guidance (Fig. 3).

**Figure 4:**
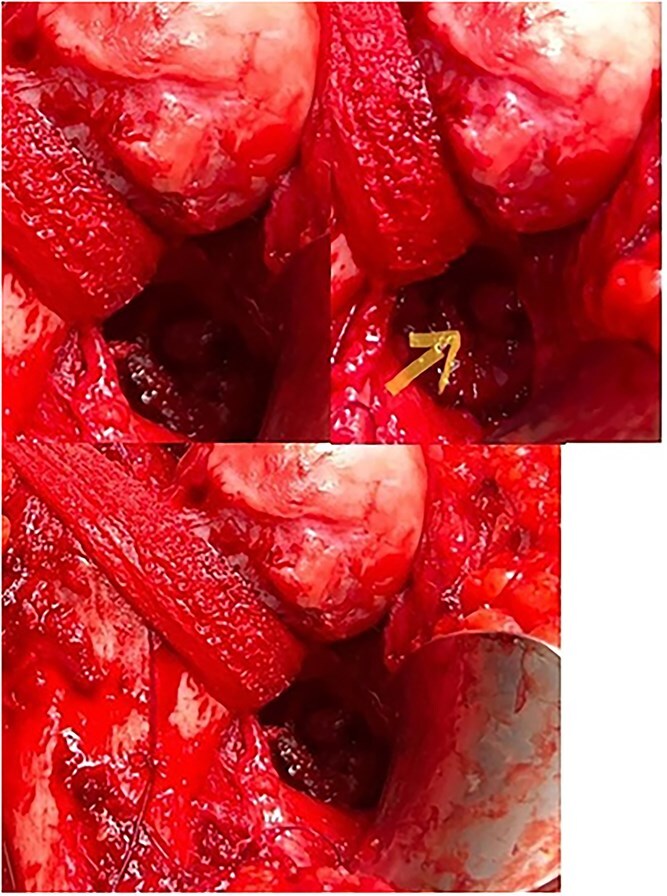
Surgical hip dislocation: Intra-acetabular view of the lesion after surgical hip dislocation.

Surgical hip dislocation allowed full 360 view of the acetabulum (Fig. 4). The lesion was identified within the central acetabular plate and completely excised using a burr until healthy bone margins were achieved. The nidus with its sclerotic border was fully removed.

**Figure 5:**
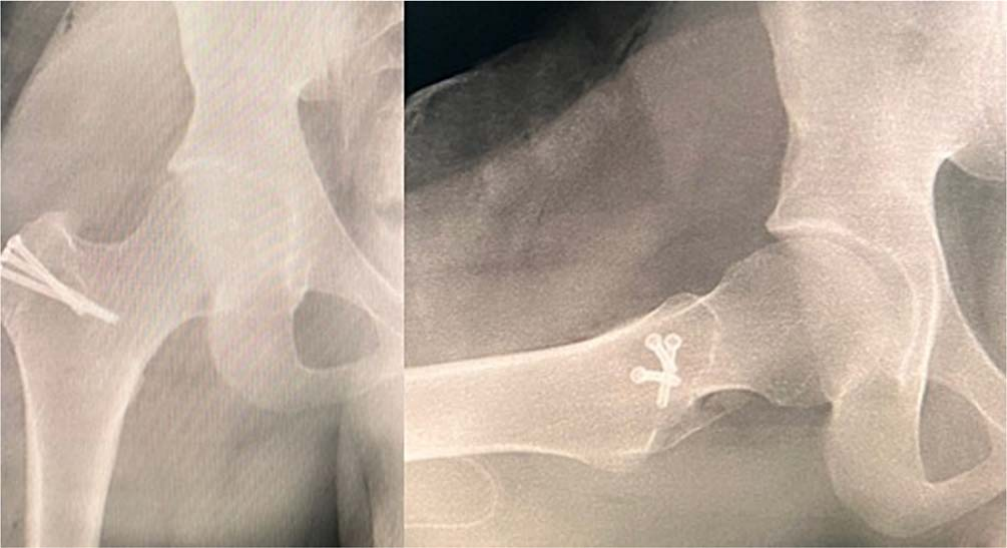
Postoperative follow-up at 6 months.

Postoperative radiographs confirmed complete excision of the lesion (Fig. 5).

The patient reported immediate resolution of nocturnal pain. At 6-month follow-up, he was completely asymptomatic, with full return to normal daily and physical activities.

## Discussion

Osteoid osteoma of the acetabulum is uncommon and remains a diagnostic and therapeutic challenge due to its juxta-articular location, nonspecific clinical presentation, and frequent overlap with other causes of hip pain. Intra-articular lesions often present with synovitis, limp, restricted range of motion, and activity-related pain, thereby obscuring the classic presentation of nocturnal pain relieved by nonsteroidal anti-inflammatory drugs. This atypical clinical picture frequently leads to delayed diagnosis and misdiagnosis as femoroacetabular impingement, inflammatory synovitis, or other intra-articular pathology. Computed tomography remains the most reliable modality for identifying the nidus, particularly in anatomically complex regions such as the acetabulum, whereas MRI may exaggerate reactive bone marrow edema and synovitis, potentially masking the nidus [1].

For most osteoid osteomas, minimally invasive CT-guided techniques have become the treatment of choice due to their high success rates and low morbidity. This also applies to many acetabular lesions, where favorable outcomes have been reported following CT-guided percutaneous resection or drilling [2, 3]. In addition, arthroscopic excision or arthroscopy-assisted ablation has emerged as a less invasive alternative for selected intra-articular lesions, demonstrating good short-term outcomes and low recurrence rates in small case series [4, 5].

Nevertheless, surgical hip dislocation retains an important role in cases where the lesion is deeply located, centrally positioned, adjacent to articular cartilage, or difficult to localize safely using percutaneous or arthroscopic techniques. The principal advantage of surgical dislocation lies in the ability to achieve direct 360° visualization of the femoral head and acetabulum, enabling precise localization and complete excision of the nidus while minimizing the risk of iatrogenic cartilage damage. The technique described by Ganz allows full access to the hip joint while preserving femoral head vascularity through a trochanteric flip osteotomy and careful protection of the medial femoral circumflex artery [6]. Early clinical series have demonstrated a negligible risk of avascular necrosis when the procedure is performed meticulously [7].

Although reports of acetabular osteoid osteoma treated with surgical hip dislocation remain limited, the available literature demonstrates consistently favorable outcomes. Karray *et al*. reported excellent results following excision through an anterior approach with hip dislocation [8]. De Los *et al*. described successful removal of an acetabular osteoid osteoma in a pediatric patient using controlled hip dislocation with gamma-probe guidance, with complete symptom resolution [9]. More recently, Tripathy *et al*. reported successful excision of a quadrilateral plate osteoid osteoma using safe surgical dislocation combined with preoperative CT planning and intraoperative fluoroscopic guidance, achieving complete resection and excellent functional recovery [10]. These findings support the role of surgical dislocation as a valuable technique for lesions located in anatomically challenging regions where minimally invasive approaches may be limited or unsafe.

In the present case, the lesion was located within the central acetabular plate and embedded in cancellous bone, rendering it difficult to access via standard arthroscopic portals or percutaneous techniques. Surgical hip dislocation provided optimal exposure, facilitated accurate fluoroscopic localization, and enabled controlled excision of the nidus and surrounding sclerotic bone under direct visualization. An additional advantage of open excision is the ability to obtain tissue for histopathological confirmation, which may be particularly important in atypical presentations or following unsuccessful prior treatment. This is clinically relevant, as incomplete removal of the nidus remains the primary cause of persistent symptoms or recurrence, regardless of the treatment modality employed.

The principal limitation of surgical hip dislocation is its invasiveness compared to CT-guided ablation or arthroscopy. The procedure requires trochanteric osteotomy, meticulous handling of retinacular vessels, and a structured postoperative rehabilitation protocol. Therefore, it should not be considered a first-line treatment for all acetabular osteoid osteomas. Instead, it should be reserved for selected cases in which lesion location, proximity to cartilage, prior failed treatment, diagnostic uncertainty, or the need for complete and controlled excision render minimally invasive techniques less suitable. In such cases, surgical dislocation represents a reliable joint-preserving option associated with excellent pain relief and low complication rates.

## Conclusion

Osteoid osteoma of the acetabulum should be considered in adolescents with persistent hip pain and inconclusive initial treatment. CT is essential for diagnosis, and surgical dislocation represents an effective treatment option for centrally located lesions.
